# Factors Associated with Road Accidents among Brazilian Motorcycle Couriers

**DOI:** 10.1100/2012/605480

**Published:** 2012-05-01

**Authors:** Daniela Wosiack da Silva, Selma Maffei de Andrade, Dorotéia Fátima Pelissari de Paula Soares, Thais Aidar de Freitas Mathias, Tiemi Matsuo, Regina Kazue Tanno de Souza

**Affiliations:** ^1^Postgraduate Program on Public Health, Department of Physiotherapy, State University of Londrina, 86038-350 Londrina, PR, Brazil; ^2^Postgraduate Program on Public Health, Department of Public Health, State University of Londrina, 86038-350 Londrina, PR, Brazil; ^3^Department of Nursing, State University of Maringá, 87020-900 Maringá, PR, Brazil; ^4^Postgraduate Program on Nursing, State University of Maringá, 87020-900 Maringá, PR, Brazil; ^5^Postgraduate Program on Public Health, Department of Statistics, State University of Londrina, 86038-350 Londrina, PR, Brazil

## Abstract

The objective of the study was to identify factors associated with reports of road accidents, among motorcycle couriers in two medium-sized municipalities in southern Brazil. A self-administered questionnaire was answered by motorcycle couriers that had worked for at least 12 months in this profession. The outcomes analyzed were reports on accidents and serious accidents over the 12 months prior to the survey. Bivariate and multivariate analyses by means of logistic regression were carried out to investigate factors that were independently associated with the outcomes. Seven hundred and fifty motorcycle couriers, of mean age 29.5 years (standard deviation = 8.1 ), were included in the study. Young age (18 to 24 years compared to ≥25 years, odds ratio [OR] = 1.77) speeding (OR = 1.48), and use of cell phones while driving (OR = 1.43) were factors independently associated with reports of accidents. For serious accidents, there was an association with alternation of work shifts (OR = 1.91) and speeding (OR = 1.67). The characteristics associated with accidents—personal (young age), behavioral (use of cell phones while driving and speeding), and professional (speeding and alternation of work shifts)—reveal the need to adopt wide-ranging strategies to reduce these accidents, including better work conditions for these motorcyclists.

## 1. Introduction

 Road accidents cause approximately 1.2 million deaths every year around the world, and there are projections that this number will grow over the coming decades, particularly in poor or developing countries [1]. In Brazil, data indicate that approximately 37,000 people die each year because of these events [2] and about 120,000 are hospitalized, considering only the hospitalizations financed by the public sector [3]. The main types of victims in Brazilian urban traffic are pedestrians and motorcyclists [2, 4].

 Studies have emphasized the role of motorcyclists in road accidents in this country from the 1980s onwards [5, 6]. Part of the growth in the number of victims that use motorcycles is attributed to a new type of work that first appeared during those years: that of motorcyclists who perform deliveries, that is, motorcycle couriers, known in Brazil as *motoboys*. Motorcycle couriers are workers who transport goods or products (e.g., documents, food, medications, mineral water, or gas bottles) or carry out small services, such as paying bills or making deposits in banks. This occupation has been seen as a work alternative for young men who do not have other job prospects [7], and a growing number of new workers has been engaging in it.

There still is a lack of studies investigating the risks to which these workers are exposed to on urban roads and to what extent they are involved in road accidents. Speeding, adverse working conditions (exposure to bad weather conditions, alternations of work shifts, and long working hours), and payment according to their performance, that is, according to the number of deliveries made are factors that may be posited to be related to accident occurrence [7, 8]. However, from reviewing the literature, it was not possible to find any studies specifically among these workers that analyzed the factors that might be associated with the risk of involvement in motorcycle accidents. Such information is essential for establishing action priorities, with the aim of avoiding these accidents. Thus, the objective of this study was to investigate the association of personal, behavioral, and professional characteristics with road accidents over the twelve months preceding the survey among motorcycle couriers in two medium-sized municipalities in southern Brazil.

## 2. Methods

 This was a cross-sectional study carried out in the municipalities of Londrina and Maringá, located in the State of Paraná, Southern Brazil, with populations of approximately 506,000 and 357,000 inhabitants, respectively [9]. The motorcycle couriers who participated in the study had been doing this type of work for at least 12 months. The data gathering took place from September to November 2005 in Londrina and from June to December 2006 in Maringá.

 Before starting to gather data, the main companies that hired motorcycle couriers were identified. These included restaurants, pharmacies, stationery stores, paint companies, gas bottle distribution companies, mineral water companies, and deliveries' companies. After telephone calls and enquiry letters sent to these companies, it was possible to determine the total number of active motorcycle couriers in both cities. Stratified random sampling was then carried out among the motorcycle couriers in Londrina, to obtain a representative sample of around 50% of the active workers in these companies. In Maringá, because of the smaller number of motorcycle couriers, all of them were approached. A self-administered anonymous questionnaire was used, consisting of both closed and open questions. This instrument was developed through stages of improvement, by assessment by researchers with expertise in epidemiology and research methodology and by means of a pilot test among motorcycle couriers who were not selected for the sample. The aim of these stages was to make the questionnaire more comprehensible for the motorcycle couriers, even for those with low educational level, and to make it quick to answer, because of the job demands of these workers.

 This research project was approved by the Research Ethics Committees of the State University of Londrina and the State University of Maringá. The motorcycle couriers were approached at their companies and were given explanations about the research objectives. They answered the questionnaire only after reading and signing the free and informed consent statement. The questionnaires were answered and placed in a sealed box to ensure anonymity among the participants. To reduce losses, up to five visits were carried out to the same company at different times of the day.

 For the electronic processing of the data, the Epi Info 6.04d software [10] was used. Double typing for data input and comparisons were carried out, and corrections were made when inconsistencies were observed between the databases, after consulting the questionnaire responses.

 The outcomes of the study were (a) reports of involvement in road accidents (yes or no) and (b) reports of involvement in serious road accidents, which were considered serious from the perception of the motorcycle couriers themselves or that required hospitalization after the accident. In both cases, only accidents that occurred over the twelve months preceding the investigation were considered, in order to reduce possible memory bias. For this reason, only data from motorcycle couriers that had been working in this profession for at least one year were analyzed, in order to standardize the length of exposure.

 The independent variables were grouped in the following manner: age (18 to 24 years; or ≥25 years); educational level (low: <8 years; or high: ≥8 years or more); length of time the individual had been driving motorcycles (<5 years; or ≥5 years); length of time working as a motorcycle courier (<3 years; or ≥3 years); payment according to performance, that is, according to the number of deliveries made (yes or no); night shift (yes or no); excessive work, that is, working ≥10 hours/day (yes or no); alternation of work shifts (yes or no); feeling of tiredness while driving (yes or no); use of cell phone or communication radio while driving the motorcycle (yes or no); consumption of alcoholic drinks before driving (yes or no); helmet model used (with or without jaw protection); speeding on city streets and in the urban area, that is, ≥80 km/h (yes or no).

For bivariate analyses, the chi-square test with Yates correction was used, or Fisher's exact test when necessary, taking a significance level of 0.05. Outcome prevalence rates (PR) were calculated (similarly to relative risks), along with their respective 95% confidence intervals (95% CI). Multivariate logistic regression with the Statistical Analysis System (SAS) software was also carried out, selecting variables by means of the stepwise method, in order to investigate factors independently associated with reports of road accidents over the previous 12 months (total of accidents and serious accidents). The criterion for including variables in the multivariate model was a *P*-value < 0.20 in the bivariate analysis, and they were kept in the model when *P* < 0.05. The Hosmer-Lemeshow goodness-of-fit test was used to analyze the fit of the models.

## 3. Results

### 3.1. Personal and Professional Characteristics of the Motorcycle Couriers

 Out of the 899 motorcycle couriers selected for the study, 877 participated in the study (loss of 2.5%). For the present analysis, 127 individuals were excluded from the sample because they had worked as motorcycle couriers for less than 12 months, resulting in a sample of 750 motorcycle couriers. Almost all of them were male (Table 1). The mean age and educational level were 29.5 years (standard deviation SD = 8.1) and 9.7 years (SD = 2.2), respectively.

About 40% of the motorcycle couriers reported working more than 10 hours a day and 30% reported alternation of work shifts. More than half reported having night shifts and payment according to the number of deliveries made. Almost 80% reported driving the motorcycle even when they were very tired and 45.2% reported speeding. These characteristics and others relating to behavior in traffic are presented in Table 1.

### 3.2. Accidents

Two hundred and seventy-eight motorcycle couriers (37.1%) reported road accidents in the 12 months preceding the survey and 82 (10.9%) were considered serious accidents.

 In the bivariate analysis, young age (18–24 years), less than 5 years of experience in driving motorcycles, less than 3 years working in this occupation, speeding, use of cell phones while driving, and use of a helmet without jaw protection were associated with reports of accidents (Table 2). Alternations, of shifts, night shifts, payment according to performance, and speeding were associated with serious accidents.

In the multivariate analysis, educational level, excessive work, night shift, payment by performance, and alcohol consumption were not included in the model for road accidents (*P* > 0.20). The same procedure was adopted for length of time driving and working, alcohol consumption, and helmet without a jaw protection for serious accidents. The factors that remained independently associated with reports of road accidents were young age (18 to 24 years), speeding, and use of cell phones while driving. Alternations of shifts and speeding were associated with serious accidents (Table 3).

## 4. Discussion

The present study aims to contribute towards expanding the knowledge about risk factors for road accidents among motorcycle couriers, thereby making it possible to identify priority intervention actions for avoiding motorcycle accidents among these workers.

However, some limitations of this study must be taken into consideration. It was not possible to identify recent cases of more severe accidents, since the motorcycle couriers who were off work due to road accidents did not participate in the survey. This may have underestimated the accident occurrence rate. Nevertheless, it must be emphasized that this study is the first Brazilian study to investigate factors associated with the occurrence of accidents among motorcycle couriers, and that the lack of records regarding such workers makes it difficult to carry out studies with other designs. Moreover, investigations on socially censured behavior may result in lower reported rates of certain types of risky behavior, such as speeding and drinking-driving. This was partially controlled for through the use of anonymous questionnaires that were filled out voluntarily, without the presence of the employers, and through ensuring confidentiality of the information, in the same way as done in other studies [11, 12].

One positive aspect of the present study is that a representative sample of active motorcycle couriers from the main types of companies offering delivery services in the municipalities analyzed was used. There was also excellent receptivity to the investigation among the motorcycle couriers themselves and a low rate of nonresponse to the questions, along with minimal losses.

The association observed in the present study between reports of accidents and the personal characteristics of the motorcycle couriers (young age) and their risky behavior in traffic (use of cell phones) was similar to findings from other investigations [6, 13]. The fact that younger individuals are more frequently involved in road accidents can possibly be explained by their greater tendency to risk taking [8, 14], along with their shorter experience of driving [6].

 The use of cell phones while driving was also a risk factor for collisions among car drivers observed at 42 points in Vancouver, Canada [15]. Cell phone use can distract drivers' attention, thereby slowing down their reaction time to risky situations and favoring the occurrence of accidents [16]. In addition, it prevents quick access by one of the hands to the vehicle's controls, which becomes even more serious when driving motorcycles because of the risk of losing balance.

The consumption of alcoholic drinks before driving vehicles is well documented in literature as a factor associated with road accidents [17–19]. However, in the present investigation, such an association was not observed. One possible reason is that the quantity of alcohol consumed and the type of drink were not investigated. In addition, the possibility of information omission relating to such behavior cannot be disregarded, as commented on earlier. This result is similar to that found in a population-based study carried out in France [20], where excessive alcohol consumption was not related to traffic accidents, probably because of a relatively high consumption of alcohol in that nation, in comparison to other countries, similarly to what occurs in Brazil [21].

 In the present study, speeding (a practice reported by 45.2% of the motorcycle couriers) was associated with both accidents and serious accidents. It was an important risk factor for the outcomes analyzed, concordant with findings from other authors [22–24]. Among the factors that explain the association between speed and accidents, the shorter time that drivers have for avoiding collisions when at high speed is emphasized. The association with serious accidents can be explained especially by the transference of kinetic energy, highly dependent on speed, to tissues, blood vessels, and other body structures of the people involved [23, 25]. Recognizing the importance of speed as a cause of accidents and injuries, the World Health Organization has launched a support manual directed to decision makers and health professionals for the adoption of speed control actions [25].

 Another contributory factor towards speeding by motorcycle couriers is the pressure from clients and employers for fast deliveries in Brazil. The pressure for rapidity and agility in a society characterized by the notion of speed and urgency legitimizes the existence of motorcycle couriers, thereby making them indispensable in large and medium-sized urban centers and for the demands of our consumer society [7, 8, 26].

It is observed, however, that despite the growing participation of motorcycle couriers in the traffic of many cities around the world, mainly in medium and low income countries [27, 28], informality in working relations predominates, with precarious working conditions, high exposure to risky situations in traffic and high rates of accidents [7, 8, 26]. It is also noteworthy the high proportion of workers who are remunerated by performance, that is, according to the number of deliveries made, which may cause higher job stress, thus increasing the probability of stress-related health problems [27, 29].

This context probably explains the association between reports of serious accidents and alternation of work shifts. A study carried out in Sydney, Australia, showed that hotel workers without formal employment links had long working hours and alternation of their work shifts, which led them to present problems such as sleep disorders, tiredness, and difficulties regarding diet and regular physical exercise [30]. Another study, among doctors in New Zealand, observed that working on night shifts and alternation of shifts were factors of greater importance in generating fatigue than was the total number of hours worked, thereby increasing the risk of accidents [31].

In addition to alternation of shifts, investigations on motorcyclists in Brazil have found reports of work overload [7, 26], including reports from motorcycle couriers that have fallen asleep while driving the motorcycle because of tiredness [7]. This leads to the need to improve the process of formalization of work relations for this professional category.

 Thus, the fact that the involvement of motorcycle couriers in road accidents or in serious accidents relates to personal characteristics (young age), to their behavior (use of cell phones in traffic and speeding), and to the rules imposed by the job market (abuse of speed and alternation of shifts) reveals the need to adopt public policies aimed towards implementing wide-ranging strategies for tackling this problem. These strategies should include improvement in the quality of motorcyclists' working conditions [8, 26], education in defensive driving for younger workers, increasing motorcyclists' conspicuity [32], and awareness-raising among employers and society in general. Most importantly, ongoing vigorous actions regarding the legislation and the environment, such as traffic calming measures and law enforcement [2, 33–35] should be put on the Brazilian political agenda with priority for preventing road accidents and, therefore, to reduce the global burden of injury.

## Figures and Tables

**Table 1:** Characteristics of the study population (*n* = 750).

Characteristic	%
Male gender	99.6
Young age (18 to 24 years)	34.1
Low educational level (1 to 7 years)	15.6
Length of time driving <5 years	18.7
Length of time working <3 years	31.3
Excessive work	40.4
Driving when tired	79.8
Alternation of work shifts	29.9
Night shift	51.5
Payment by performance	56.2
Speeding	45.2
Use of cell phone while driving	23.1
Alcohol consumption before driving	39.1
Use of helmet without jaw protection	44.7

**Table 2:** Association between characteristics of the motorcycle couriers and reports of road accidents and serious accidents: prevalence ratio, PR (95% CI).

Variables	Accidents		Serious Accidents	
	PR	95% CI	PR	95% CI
Young age (18 to 24 years)	1.49*	(1.24–1.79)	1.12	(0.83–1.51)
Low educational level (1 to 7 years)	1.02	(0.79–1.32)	1.39	(0.87–2.28)
Length of time driving <5 years	1.30***	(1.06–1.61)	1.13	(0.72–1.77)
Length of time working < 3 years	1.33**	(1.11–1.61)	1.10	(0.80–1.52)
Excessive work	1.06	(0.87–1.28)	1.24	(0.97–1.58)
Driving when tired	1.27	(0.98–1.65)	1.08	(0.98–1.19)
Alternation of work shifts	1.20	(0.99–1.46)	1.58**	(1.20–2.07)
Night shift	0.89	(0.74–1.07)	1.27***	(1.06–1.52)
Payment by performance	1.03	(0.86–1.25)	1.25***	(1.06–1.47)
Speeding	1.39*	(1.15–1.67)	1.31***	(1.07–1.61)
Use of cell phone in traffic	1.37**	(1.13–1.67)	1.38	(0.96–1.97)
Alcohol consumption	1.10	(0.91–1.33)	0.93	(0.69–1.25)
Use of helmet without jaw protection	1.24***	(1.03–1.49)	1.17	(0.93–1.47)

*P < 0.001; **P < 0.01; ***P < 0.05.

**Table 3:** Multivariate analysis for reports of road accidents and serious road accidents.

Characteristics	OR	CI 95%
*Accident* ^ a^		
Age 18–24	1.77*	(1.29–2.44)
Speeding	1.48***	(1.08–2.03)
Use of cell phone in traffic	1.43***	(1.00–2.06)
*Serious accident* ^ b^		
Alternation of work shifts	1.91**	(1.18–3.06)
Speeding	1.67***	(1.04–2.68)

*P < 0.001; **P < 0.01; ***P < 0.05.

^a^ *Hosmer-Lemeshow goodness-of-fit test: X^2^ = 3.3608; 5 degrees of freedom;* *P* = 0.6445.

^b^ *Hosmer-Lemeshow goodness-of-fit test: X^2^ = 2.0716; 2 degrees of freedom;* *P* = 0.3549.
